# Depression, anxiety, PTSD, and OCD after stillbirth: a systematic review

**DOI:** 10.1186/s12884-021-04254-x

**Published:** 2021-11-18

**Authors:** Cèline Lossius Westby, Andrea Røsberg Erlandsen, Sondre Aasen Nilsen, Endre Visted, Jens C. Thimm

**Affiliations:** 1 Centre for Crisis Psychology, Faculty of Psychology, University of Bergen, 5020 Bergen, Bergen, Norway; 2 Regional Centre for Child and Youth Mental Health and Child Welfare, NORCE Norwegian Research Centre, Bergen, Norway; 3 Department of Health Promotion and Development, Faculty of Psychology, University of Bergen, Bergen, Norway; 4 Department of Clinical Psychology, University of Bergen, Bergen, Norway; 5 Department of Psychology, Faculty of Health Sciences, UiT The Arctic University of Norway, Tromsø, Norway

**Keywords:** Stillbirth, Depression, Anxiety, PTSD, OCD, Parents, Systematic review

## Abstract

**Background:**

This systematic review aimed to provide an updated summary of studies investigating depression, anxiety, post-traumatic stress disorder (PTSD), and obsessive-compulsive disorder (OCD) in parents after stillbirth (from 20 weeks gestational age until birth).

**Methods:**

A literature search was conducted in the databases Web of Science and PsychINFO. Main inclusion criteria were 1) peer-reviewed, quantitative, English-language articles published from 1980; (2) studies investigating depression, anxiety, PTSD, or OCD among parents following stillbirth; and (3) studies defining stillbirth as equal to or after 20 weeks of gestation.

**Results:**

Thirteen quantitative, peer-reviewed articles were eligible for inclusion. Selected articles investigated depression, anxiety, and PTSD, while no studies on OCD met our inclusion criteria. The majority of studies investigated women, while only two studies included men. The results indicated heightened short- and long-term levels of depression, anxiety, and PTSD in parents after stillbirth compared to those of parents with live birth. Studies investigating predictors found that social support, marital status, negative appraisals, and variables related to care and management after stillbirth affected levels of symptoms.

**Conclusions:**

Parents who experience stillbirth have a considerably higher risk of reporting symptoms of depression, anxiety, and PTSD compared with parents with live births. More longitudinal studies are needed to increase our knowledge of how symptoms develop over time, and more research on fathers, transgender, non-binary and gender fluid individuals is needed. Research on the association between stillbirth and OCD is also warranted. Knowledge of the severity of anxiety, depression, and PTSD after stillbirth, and predictors associated with symptom severity could provide healthcare professionals with valuable information on how to provide beneficial postpartum care.

**Supplementary Information:**

The online version contains supplementary material available at 10.1186/s12884-021-04254-x.

## Background

Stillbirth (SB) is commonly defined as in utero fetal death or death at birth when the fetus is ≥20 weeks of gestation (GSA) [1–5]. However, various definitions exist, with numerous SB classification systems in the literature [6, 7]. This makes global prevalence rates difficult to report. Still, available estimates suggest that 1.9 million stillbirths ≥28 GSA occurred globally in 2019 [8], equalling approximately 5200 incidents on any given day. The prevalence varies significantly between high- and low−/middle-income countries (LMICs), as 98% of stillbirths occur in the latter [9].

SB is associated with various adverse psychosocial outcomes in parents [10]. In contrast to early pregnancy loss (e.g., miscarriage), SB can occur close to full-term [11], and often without previous signs or obvious reasons [12]. As noted by Bennet et al. [13], SB involves the loss of a child never met or known, and the loss of a possible future. Parents may have spent months preparing for the infant’s birth, leaving them shocked to learn of their child’s death [14, 15]. In parallel, parents can feel alone, as others might lack the understanding that their loss is significant, or avoid talking about the stillborn [13]. The potential absence of social support can contribute to perceiving one’s emotional reactions as invalid or shameful [16], which may intensify the mourning process [17, 18].

Stress, anxiety, and depression symptoms are common immediate reactions to SB [17, 19]. However, for some parents, these symptoms may become pathological and long-lasting [10, 20, 21]. For example, two longitudinal studies found significantly more symptoms of depression and anxiety in mothers and fathers when assessed 8 months after stillbirth compared to parents with live birth infants [2, 5]. This difference remained relatively stable at 2.5-year follow-up [2]. In a study by Gravensteen et al. [22], one-third of women with previous SB reported post-traumatic stress disorder (PTSD) symptoms above clinical cut-off when assessed 5–18 years after SB. SB is also found to increase the risk of depression and anxiety symptoms in subsequent pregnancies [23]. This review aimed to provide an updated summary of studies investigating depression, anxiety, PTSD, and obsessive-compulsive disorder (OCD) after SB, and to explore predictors associated with symptom severity.

### Previous reviews on stillbirth and mental health

Several literature reviews have attempted to synthesize the existing empirical knowledge on psychological outcomes after SB [10, 14, 24–28]. Overall, these reviews report adverse short- and long-term psychological outcomes in parents following SB. For example, Campbell-Jackson and Horsch [24] found high levels of anxiety, depression, and distress, with the highest levels of symptoms in the first months after SB. Still, several methodological aspects of existing work suggest that an updated review is warranted. First, the most recent review on parents’ mental health after SB included studies published up until February 2015 [10]. Thus, no other reviews have included studies published in the last 6 years.

Second, existing reviews have often included a broad range of psychological outcomes. For example, Burden et al. [10] examined more than 20 different psychological outcomes, including disenfranchised grief, avoidance of memories, altered body image, and chronic pain and fatigue. Similarly, another review included additional measures of grief, drug and alcohol use, marital satisfaction, and well-being [24]. Although providing a comprehensive overview of the topic, broad inclusion criteria may preclude a more detailed assessment of specific symptomatology. To our knowledge, no other reviews have solely focused on depression, anxiety, PTSD, and OCD following SB.

Third, most of the reviews have included both qualitative and quantitative research and paid less attention to the numerical estimate of the associations between SB and parental mental health from quantitative research. The latter could provide an overview of the prevalence, severity, and predictors of mental health problems, which may inform future research and illuminate the needs of parents experiencing SB.

Lastly, no reviews to date have included OCD as an independent outcome-measure. As major transitional periods, including pregnancy and childbirth, have been associated with the onset or exacerbation of OCD symptoms [29], a systematic review of research examining the association between SB and OCD is warranted.

### The present review

This review aimed to provide a systematic overview of the existing body of quantitative research on anxiety, depression, PTSD, and OCD in parents following SB. An updated assessment of currently available evidence may increase awareness of the psychosocial impacts of SB. Further, a summary could provide a base for future research on possibly neglected psychological aspects of parental health after SB. As both parents’ mental health is presented in this review, possible differences between genders could be identified. A systematic review could also inform or improve bereavement care, preventive interventions, and healthcare guidelines, with the potential to alleviate some of the emotional burden associated with SB experiences.

Based on the above considerations, the study objectives were to identify (1) the severity of anxiety, depression, PTSD, and OCD in parents after SB, and (2) predictors associated with symptom severity among parents following SB. Thus, we sought to answer the research questions: (1) do parents who have experienced SB have higher levels of depression, anxiety, PTSD, and/or OCD compared to parents who had live births?; and (2) what predictors are associated with the severity of symptoms of depression, anxiety, PTSD, and OCD after SB?

## Methods

### Study design

We conducted a systematic review of peer-reviewed, quantitative studies, evaluating levels and predictors of depression, anxiety, PTSD, and/or OCD symptoms in parents after experiencing SB. SB was defined as fetal death at ≥20 gestational weeks until birth. The Preferred Reporting Items for Systematic Reviews and Meta-Analyses (PRISMA) statement [30] was used as guidance for reporting. The systematic review was conducted according to a pre-planned protocol agreed upon by the first and senior authors.

### Data sources and search strategy

**Fig. 1:**
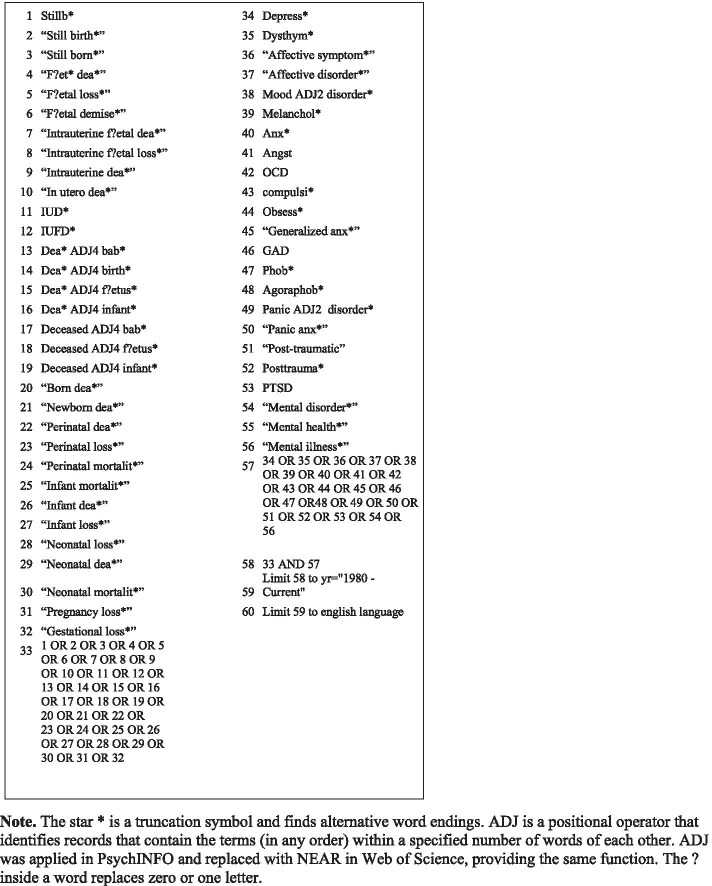
Search strategy in PsycINFO and Web of Science.

An electronic keyword search in two online databases (PsychINFO and Web of Science) was conducted on August 28th, 2020. A comprehensive search strategy was developed prior to the search. The search terms and combinations applied are detailed in Fig. 1. Initial exploratory searches in the two databases were conducted to identify keywords of interest to inform our search strategy. The final search strategy was implemented identically in both databases. Finally, reference lists from former studies and reviews were carefully read to include potentially eligible records.

### Inclusion and exclusion criteria

Inclusion criteria were: (1) peer-reviewed, quantitative, English-language articles published from 1980; (2) studies investigating at least one of the targeted mental health outcomes (i.e., depression, anxiety, PTSD, or OCD) experienced by one or both parents following SB with or without a control group (i.e., parents experiencing live birth); and (3) studies providing a clear definition of SB as equal to or after 20 weeks of gestation.

Articles were excluded if (1) the women were currently pregnant subsequent to a previous loss, due to the possible influence that a subsequent baby could have on parental mental health. For instance, studies suggest that depression and anxiety may be prevalent and prolonged in the pregnancy following SB and that pregnant women may be more vulnerable to depression and anxiety [23, 31].

We did not include studies investigating (2) elective abortions after the 20th week of gestation due to fetal anomalies, as the parents may actively had to decide whether or not the baby should be kept. Parents may have different psychological reactions in pregnancies with known fetal abnormalities or complications, as they are informed of the fetus’s health condition and thus can prepare for the loss [32]. Articles were also excluded if (3) the sample included twin births with two stillborns or if the stillborn baby had a live-born twin. In the case of stillborn twins, parents might experience more severe symptoms of anxiety, depression, PTSD, or OCD, due to a double loss. On the contrary, parents could experience less symptomatology if one twin survives. In addition, parents might be aware of the heightened risk of complications in multiple births (i.e., birth of two or more offspring), which could leave them more prepared for loss than parents with singleton births. Furthermore, we excluded (4) intervention studies and case studies, articles without primary data (systematic reviews and meta-analysis), and articles that did not isolate results for SB from other forms of perinatal loss (i.e., miscarriage, neonatal death, and sudden infant death syndrome). Lastly, (5) dissertations, letters, conference abstracts, and editorials were excluded.

### Data analyses

Search results were transferred to the internet-based program Rayyan QCRI [33] after duplicates were removed. The titles and abstracts were independently evaluated for eligibility by two investigators (AE, CW). Discrepancies in decisions were resolved by discussion. Full text articles were also screened separately, followed by a reconciliation process between investigators. Data from the included studies were extracted seperately by AE and CW using a constructed template for the purpose and compared before inclusion. Information extracted for qualitative synthesis were author names, publication year, country, study design, definition of SB, number and age range of participants, time of measurement, outcome (type of outcome and measures), covariates, and quantitative indices of group differences (e.g., risk ratios or odds ratios). When available, adjusted estimates were prioritized.

### Quality assessment

The quality of included studies was assessed using the quality checklist for quantitative studies proposed by Kmet and colleagues [34]. Two authors (CW and AE) independently assessed the studies, and disagreements were solved by consensus. Agreement ratings were not recorded. Studies were scored on a three-point Likert-scale (0 = no, 1 = partial, 2 = yes) on 11 criteria. The sum of all scores was then divided by the highest possible score (22), providing quality scores ranging from 0 (worst) to 1 (best).

## Results

### Study selection

**Fig. 2:**
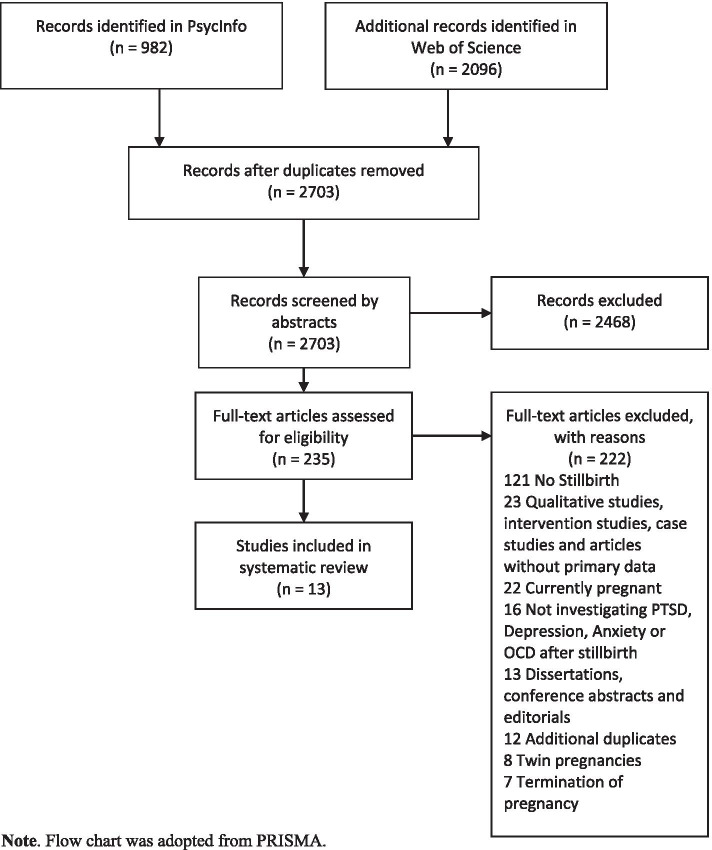
Flow chart of study selection

The final results of our systematic literature search identified 3078 potentially eligible studies. After removing duplicates, the final number of records was 2703. Screening of titles and abstracts resulted in 235 articles further investigated in full text. A total of 13 studies were selected for inclusion in the final analysis (see Fig. 2, for flow chart). The present review includes five new studies published between 2015 and 2020 that have not been included in previous reviews [35–39]. No eligible studies were included from reference lists from former studies and reviews. Due to the relatively small number of studies identified and large heterogeneity between studies in terms of study design and measurement points, the data were not suited for meta-analysis.

### Description and quality assessment of the included studies

Characteristics of the included studies are shown in Table 1. The articles were published between 1995 and 2019. The majority of studies were conducted in Europe (*n* = 8), followed by Australia (*n* = 3),USA (*n* = 1) and Bangladesh (*n* = 1). Most studies utilized a cross-sectional design (*n* = 3) or linked data from different registries (*n* = 6), three studies had longitudinal data, and one study conducted secondary analysis on data from a randomized controlled study. Seven of the 13 studies included a control group of parents who had experienced live birth (hereinafter *control group* or *controls*). Across the studies, the number of participants that experienced SB varied from 50 to 8292, with control group samples consisting of 50 to 1,194,758, accordingly. Parental age ranged from 13 to 54 years. However, seven studies only reported the mean age of participants (range 27.4–35.9 years). Only two studies included male participants in addition to women. Assessments mostly took place between 2 months and 3 years after SB, whereas three studies only provided the mean time since SB. The studies differed in the numbers of reported mental health outcomes. Three articles investigated anxiety, depression, and PTSD after SB, five studies examined depression and anxiety, four only reported depressive symptoms, and one solely explored PTSD. We did not find any studies on OCD after SB that met our inclusion criteria. Most studies (*n* = 9) were rated to be of moderate to high quality (range 0.81–1), while four studies had a lower quality score (ranging 0.59–0.77). Small sample sizes and incomplete baseline and demographic information were the most common reason for low quality scores across the studies (see Supplementary Table 1).

**Table 1:** Study characteristics of included studies

First author	Country	Study design	Definition SB	Participants	***N***	Age range	Time of measure	Outcome	Measures	Covariates/ matched control	Quality score
Boyle (1996) [2]	Australia	Longitudinal case control study	≥20 weeks	Women who had experienced SB, NND or SIDS between 1985 and 1988	SB = 78 Controls = 203	N/D	2-, 8-, 15- and 30-months post-loss	Anxiety and depression	DSSI	**Covariates:** Maternal age, marital status, other children **Matched control:** on child’s sex, hospital of delivery, birth date, health insurance status.	0.86
Chung (2017)[35]	UK	Cross-sectional retrospective cohort study	≥24 weeks	Women recruited through two SB support groups	SB = 50 Controls = 50	SB group M = 35.86 Comparison group M = 33.10	3.5 years post-stillbirth (M = 44.76 months, SD = 33.16 months)	Depression, anxiety, PTSD	EPDS, GHQ-28, PDS (PTCI)	**Covariates:** Marriage, ethnicity, education, income	0.90
Crawley (2013) [3]	UK	Cross-sectional retrospective online survey	≥20 weeks	Women who experienced SB in the past 10 years, recruited through support websites	162	18–47	0–10 years (M = 27.9 months)	Depression, anxiety, PTSD	DASS-21, PSS	None	0.90
Horsch (2015) [36]	UK	Short-term longitudinal Study	≥24 weeks	Women who had experienced SB, contacted through their bereavement midwife at eight National Health Service Hospitals in England	65	> 18 years (M = 31.92)	3- and 6-months post-loss	PTSD	SCID - PTSD module, PDS, (PTCI)	**Covariates:** Social support, maternal age, income, living children, history of trauma, history and number of previous perinatal loss, pregnancy planned, weeks of gestation.	0.86
Kokou-Kpolou (2018) [37]	France	Cross-sectional questionnaire study	≥28 weeks	Women recruited through groups for bereaved individuals on Google	SB = 66	20–49	M = 40.48 months (SD = 28.92, range 3–120)	Depression	CESD-R-10	**Covariates:** Age and time since perinatal loss	0.81
Lewkowitz (2019) [38]	USA	Retrospective cohort study	≥23 weeks	Female Florida-residents identified through The Florida State Inpatients and State Emergency Department databases	SB = 8292 Controls = 1.194.758	13–54	Within 1 year after hospital discharge	Depression, anxiety, PTSD	ICD-9-CM	**Covariates:** Maternal age, race/etnicity, insurance type, income quartile by zipcode, severe intrapartum maternal morbidity, and medical comorbidities.	1
Rådestad (1996) [40]	Sweden	Nationwide cohort study (NBHW)	≥28 weeks	Women who experienced SB in 1991, identified through the medical birth register	SB = 380 Controls = 379	M = 32	3 years post-loss	Anxiety and depression	STAI, CES-D	**Covariates:** Age, level of education, being single, being unemployed, no subsequent pregnancy	0.72
Rådestad (2009) [41]	Sweden	Nationwide cohort study (NBHW)	≥28 weeks	Women who experienced SB in 1991, identified through the medical birth register	314	M = 32	3 years post-loss	Anxiety and depression	STAI-S, STAI-T and CES-D	None	0.77
Surkan (2008) [42]	Sweden	Nationwide cohort study (NBHW)	≥28 weeks	Women who experienced SB in 1991, identified through the medical birth register	314	M = 32	3 years post-loss	Depression	CES-D	**Covariates:** Maternal education, employment, marital status	0.90
Surkan (2009) [43]	Sweden	Nationwide cohort study	≥28 weeks	Women who experienced SB in 1991, identified through the medical birth register	298	M = 32	3 years post-loss	Depression	CES-D	**Covariates:** Age, education, marital status, employment, religion, child gender, GSA, birth weight	0.90
Surkan (2016) [39]	Bangladesh	Retrospective study	≥28 weeks	Married women identified through JiVitA-1study between 2001 and 2007 in Northwest Bangladesh	SB = 1914 Controls = 37.080	13–44	6 months post-loss	Depression	Modified questionnaires based on Patient Health questionnaire and CES-D (translated into Bengali)	**Covariates**: Maternal age, education, parity, household living standard index, religion, number of children in household, maternal mid-upper arm circumference in third trimester, symptoms of anemia, and infection in the first trimester	0.90
Thearle (1995) [4]	Australia	Matched case control study (prospective)	≥20 weeks	260 families (including men and women) who experienced SB, NDD, or SIDS in Queensland between 1985 and 1988	SB mothers = 99 Control mothers = 249 SB fathers = 80 Control fathers = 204	Mothers M = 27.4 Fathers M = 30.2	2 months post-loss	Anxiety and depression	DSSI (subscales depression and anxiety)	**Matched control:** child of same sex born at the same hospital on the same date as the stillborn child, same health insurance status.	0.59
Vance (1995) [5]	Australia	Longitudinal case control study	≥20 weeks	Families (including men and women) who experienced SB, NDD, or SIDS in Queensland between 1985 and 1988	SB mothers = 82 Control mothers = 226 SB fathers = 65 Control fathers = 174	Mothers M = 27.4 Fathers M = 30.2	2- and 8-months post- loss	Anxiety and depression	DSSI (subscales depression and anxiety)	**Matched control:** child of same sex born at the same hospital on the same date as the stillborn child, same health insurance status.	0.72

Note. *SB* Stillbirth, *NND* Neonatal death, *SIDS* Sudden infant death, *N/D* No data, *DSSI* Delusions Symptoms States Inventory, *UK* United Kingdom, *SD* Standard Deviation, *M* Mean, *EPDS* Edinburgh Post-natal Depression Scale, *GHQ-28* General Health Questionnaire-28, *PDS* Posttraumatic Diagnostic Scale, *PTCI* Posttraumatic Cognitions Inventory; *DASS* Depression, Anxiety, and Stress Scale, *PSS* Posttraumatic Stress Symptom Scale, *SCID* Structured Clinical Interview-DSM-IV-PTSD module, *ICD-9-CM* International Classification of Diseases, 9th Revision, *STAI* State Trait Anxiety Inventory, *CES-D* Center for Epidemiological Studies Depression Scale

The results of the systematic review are presented in the following by mental disorder. Within each outcome measure, we present findings from studies investigating predictors of symptom severity among parents who experienced SB.

### Depression

#### The severity of depression after stillbirth

Twelve studies investigated depressive symptoms in parents with SB. Seven of these studies included a control group of parents with live births; three were retrospective cohort studies [38–40], one study utilized a cross-sectional design [35], and one investigation applied a prospective case-control design [4]. The remaining two studies were longitudinal case-control studies [2, 5]. Depression was assessed from 2 months to 3.5 years after SB (for further study details, see Table 1).

All studies reported a significant association between SB and parental depression symptoms after controlling for SES. However, the strength of the association varied. For example, Chung and Reed [35] found that mothers who experienced SB had a significantly higher adjusted odds ratio of depression (*OR*_*adj*_ = 5.34 [95% CI 2.15–13.29]) than controls 3.5 years after the event. Another study found that the odds of depression (as classified by the International Classification of Diseases, 9th Revision [44];) was 2.75 (95% CI 1.93–2.70) among mothers who had been hospitalized or in contact with an emergency department within 1 year of SB, compared to controls [38]. Thearle et al. [4] found significantly more symptoms of depression in both mothers and fathers compared to those in matched control groups.

The two longitudinal case-control studies compared mothers [2] or mothers and fathers [5] who had experienced SB with matched controls who had experienced live birth. For mothers, the studies found a higher risk of depression at 2- and 8 months follow-up, compared to controls. However, the risk estimates were higher at both time-points in the study by Vance et al. [5]. The RR was 6.70 (95% CI 2.90–15.60) at 2 months, before declining to RR = 2.40 (95% CI 0.83–6.90) at 8 months [5]. Boyle et al. [2] found a RR of 5.49 (95% CI 2.33–12.96) and RR = 1.74 (95% CI 0.50–6.00), respectively. Boyle et al. [2] further assessed depression at 15- and 30 months follow-up. The results showed a slight increase from 8 to 15 months (RR = 2.65 [95% CI 0.68–10.32]), and from 15 to 30 months follow-up (RR = 2.78 [95% CI 0.83–9.33]).

For fathers, Vance et al. [5] found a substantially higher risk of high depression score 2 months after stillbirth compared to controls (RR = 5.90 [95% CI 1.20–94.00]), a risk that remained stable at 8 months follow up (RR = 6.10 [95% CI 1.20–94.00]).

Overall, the findings suggest that SB is associated with depression symptoms. The longitudinal studies indicate that the highest risk of depression presents shortly after birth and although the symptoms decline with time, they remain higher than among controls. Although no *p-*values were provided in these studies, the reported confidence intervals were wide, and crossed the value 1 at 8 months and at later follow-ups for mothers. Thus, these reported risks were not statistically significant at an alpha of .05. The wide confidence intervals further suggest high uncertainty in the point estimates. Thus, some caution should be applied when interpreting these results.

#### Predictors of depression after stillbirth

Six studies investigated predictors of depression symptoms among women who had experienced SB [3, 37, 40–43]. Identified predictors were marital status, educational status, time since SB, professional and social support, negative cognitions (e.g., “I am worthless”, “the world is unjust”), no future pregnancies, not having had the desired contact with the stillborn, birth order of the stillborn, and whether mothers experienced difficulty in becoming pregnant. Overall, most predictors had a weak to moderate influence (RRs ranging 1.30–2.80) on depression symptoms. Across all studies, being unmarried (versus married) was associated with the highest risk of depression among women who had experienced SB, even after adjusting for educational qualifications and employment status (RR = 9.80 [95% CI 5.40–18.00]) [43]. The second highest risk factor was reported dissatisfaction with the emotional support received (RR = 8.30 [95% CI 1.60–42.00]), after controlling for maternal education, employment, and marital status [43]. Another study by Surkan et al. [42] (utilizing the same data) identified not being with the baby for as long as desired (*RR*_*adj*_ = 6.90 [95% CI 2.40–19.80]) and occurrence of the SB in the fourth or later pregnancy (*RR*_*adj*_ = 6.70 [95% CI 2.20–20.50]) as significant risk factors of maternal depressive symptoms.

### Anxiety

#### The severity of anxiety after stillbirth

Eight studies investigated severity of anxiety symptoms among parents who had experienced SB. Six of these studies included a control group; two were retrospective cohort studies [35, 38], one utilized a cross-sectional design [36], and one applied a case-control design [4]. The remaining two were longitudinal case-control studies [2, 5]. Anxiety was assessed from 2 months to 3.5 years after SB (for further study details, see Table 1).

The study by Chung and Reed [35] found that mothers who had experienced SB had significantly higher anxiety mean scores than controls. Thearle et al. [4] found significantly more symptoms in both mothers and fathers than in controls. However, mothers reported more symptoms of anxiety than fathers [4].

Rådestad et al. [40] found a two-fold risk (RR = 2.10 [95% CI 1.20–3.90]) of anxiety symptoms in women who experienced SB compared to women with live birth. However, no information on adjustments made for this estimate was given. In the study by Lewkowitz et al. [38], the odds of being coded for anxiety according to the International Classification of Diseases, 9th Revision, during an Emergency Department encounter or inpatient admission within 1 year of delivery, was *OR*_*adj*_ = 2.29 (95% CI 1.93–2.70), when adjusted for several control variables (Table 1).

Two longitudinal case-control studies assessed anxiety in parents following SB [2, 5]. Boyle et al. [2], found that women with SB had a RR = 4.34 (95% CI 2.30–8.18) of scoring above cutoff for anxiety at 2 months compared to women with live birth. At 4 months follow up, the risk declined to RR = 2.61 (95% CI 1.18–5.77). Vance et al. [5] found a RR of 5.10 (95% CI 2.70–9.50) for women with SB compared to mothers with live birth at 2 months and an RR of 3.0 (95% CI 1.40–6.40) at 8 months follow up. Fathers had close to a five-fold risk (RR = 4.70 [95% CI 1.40–15.50]) of anxiety at 2 months compared to fathers with live birth, a risk that remained high at 8 months follow up (RR = 4.0 [95% CI 1.20–13.80]) [5]. Boyle et al. [2] further assessed anxiety at 15 and 30 months follow-up. The results showed a minimal increase from 8 to 15 months (RR = 2.65 [95% CI 1.15–6.10]), before slightly declining at 30 months follow up (RR = 2.09 [95% CI 0.92–4.74]).

Overall, the findings suggest that SB is associated with anxiety symptoms in the postpartum period, and that parents experiencing SB have a heightened risk compared to parents with live birth. The longitudinal studies indicate that the highest risk of anxiety presents shortly after birth. Although the risk declined with time, particularly at 30 months follow up, the risks remained observably higher than among controls. Of note, the risk at 30 months appeared to be not statistically significant (at alpha = .05), as the reported confidence interval contained the value 1 [2].

#### Predictors of anxiety after stillbirth

Three studies [3, 40, 41] investigated predictors of anxiety following SB. The predictors included time since given diagnosis of death until the start of birth, seeing the child as long as desired, having tokens of remembrance, holding the stillborn after birth, time since SB, and the wish to talk more about the baby.

Rådestad et al. [40] found a higher prevalence of anxiety in women that had to wait more than 25 h after diagnosis of death in utero until the onset of birth, compared to women with less delay. In total, 23% of women with delay had scores above the 90th percentile on The State-Trait Anxiety Inventory (STAI [45];), in contrast with 5% of women with no delay (RR = 4.60 [95% CI 1.50–15.90]). Similarly, women who did not see their baby as long as desired had a higher prevalence (19%) of anxiety than women who did not report this (8%; RR = 2.4 [95% CI 1.10–5.30]). Women with no token of remembrance (i.e., photo of the baby, a lock of hair, handprints, or footprints) reported more anxiety than women who collected tokens (RR = 4.00 [95% CI 1.40–11.30]), with prevalence rates of 22 and 7%, respectively. Utilizing the same sample, a later study by Rådestad et al. [41] investigated whether having held the stillborn or not was associated with levels of anxiety. The study did not detect a significant relationship between whether the mothers held the stillborn and anxiety symptoms.

Crawley et al. [3] found that time passed since SB was negatively associated with anxiety scores. Mothers who wished to talk more about the baby reported more anxiety symptoms than mothers who did not report this wish. These two variables combined accounted for 7.7% of the variance in anxiety scores.

### PTSD

#### The severity of PTSD after stillbirth

Two cross-sectional studies [3, 35], one short-term longitudinal study [36], and one retrospective cohort study [38] investigated symptoms of PTSD following SB (see Table 1 for study details). Overall, the studies reported a significant association between having experienced SB and PTSD, three of them after controlling for SES [35, 36, 38].

Crawley et al. [3] found elevated mean scores of PTSD on the Posttraumatic Stress Symptom Scale (PSS) in mothers who had experienced SB. In the study by Lewkowitz et al. [38], the risk of being diagnosed with PTSD 1 year after SB was two times higher than the risk of being diagnosed with depression and anxiety. Mothers with SB had above four times higher risk (RR = 4.36 [95% CI 2.31–8.24]) than women with live birth to receive a PTSD diagnosis, after adjusting for SES, mode of birth, intrapartum maternal morbidity and medical comorbidities. Chung and Reed [35] found that 60% of their sample met the diagnostic criteria for PTSD when assessed with the Posttraumatic Diagnostic Scale (PDS [46]).

In the short-term longitudinal study, measurements were obtained at 3- and 6 months after SB [36]. Symptoms of re-experiencing (e.g., flashbacks and intrusive thoughts), avoidance behavior, and arousal (e.g., difficulty concentrating and hypervigilance) were highest at 3 months after SB, before declining at 6 months (the decline in symptoms corresponded to a reduction in Cohen’s *ds* of 0.34–0.52).

Summarized, the findings indicate that mothers exposed to SB are more likely to develop symptoms of PTSD and have a higher prevalence of diagnosed PTSD than mothers with live birth. The symptoms appear to be highest in the immediate postnatal period, followed by a decline as time passes.

#### Predictors of PTSD after stillbirth

Three studies [3, 35, 36] investigated various predictors of PTSD among parents who experienced SB. These predictors included cognitive factors (i.e., appraisals, dysfunctional strategies, locus of control, and posttraumatic cognitions), perceived social and professional support, time since SB, and memory-making activities.

Crawley et al. [3] identified professional support, time since the baby died, how memories were shared, and the wish to talk more about the stillbirth as strong predictors of PTSD, accounting for 24.4% of the variance in PTSD symptoms. Specifically, their findings suggested that an increase in professional support (*B* = − 2.63), in sharing of memories (*B* = − 0.63), and in time since the baby died (*B* = − 0.14) were significantly associated with a decrease in PTSD symptoms. On the other hand, the extent to which they expressed the wish to have talked more about the baby was significantly associated with an increase in PTSD symptoms (*B* = 2.09). Horsch and colleagues [36] found that rumination and negative appraisals (negative cognitions about the self and the world) were significantly associated with both frequency and number of PTSD symptoms. Additional risk factors for PTSD symptoms included younger age, lower income, and no previous pregnancies. That is, having more children and older age in mothers predicted fewer avoidance symptoms at 6 months, whereas a higher income was associated with a decline in arousal symptoms. Women who rated perceived social support as high reported lower levels of re-experiencing symptoms at 3- and 6 months, indicating social support as a protective factor for PTSD [36].

## Discussion

### Summary of findings

The present systematic review sought to summarize quantitative studies examining the associations between SB and symptoms of parental depression, anxiety, PTSD, and OCD. Embedded within this goal, we also aimed to synthesize the existing knowledge of risk- and protective factors for these symptoms following SB. Most of the studies investigated depression (*n* = 12) or anxiety (*n* = 8), while 4 studies examined PTSD. We found no eligible studies investigating OCD.

Overall, the body of research systematically reviewed indicates that SB is associated with short- and, in part, long-term levels of depression, anxiety, and PTSD in parents. This conclusion appears rather consistent across study design, participant characteristics, and assessment instruments. The cross-sectional studies found more symptoms in parents when assessed up to 3.5 years after SB compared with controls. The longitudinal studies generally found a higher risk of symptoms compared with controls at first assessment after stillbirth. However, the difference between the stillbirth group and control group was not significant at 8 months later and at subsequent follow-ups for depression. Similarly, the risk of anxiety was not significantly different at the 30-months follow-up. Thus, while both cross-sectional and longitudinal studies indicate potential long-term consequences for parents after SB, the longitudinal studies are not able to draw clear conclusions regarding parents’ long-term levels of depression and anxiety after experiencing SB.

The included studies examined various predictors of depression-, anxiety-, and PTSD symptoms following SB. The predictors included both aspects of healthcare and intra- and interpersonal factors. For example, having to wait more than 25 h after diagnosis of death in utero until birth was associated with heightened anxiety levels. Satisfaction with social support and being married predicted less depression symptoms, while rumination and negative cognitions about the self and world were associated with more PTSD symptoms.

### Interpretation of findings

Overall, studies with control groups indicate SB as a predictor of later depression, anxiety, and PTSD. However, the strength of associations varied between the studies (RRs ranged from 1.74 to 6.70). This may be due to differences in sample sizes, psychometric properties of the instruments utilized, and assessment time in the studies.

A general trend was observed across studies, whereby small-sample studies tended to report the largest effect sizes. Small-sample studies are generally more prone to bias, and the relatively large confidence intervals presented in these studies suggest high uncertainty around the point estimates [47, 48]. The results of these studies should, therefore, be interpreted with some caution.

Furthermore, eight different instruments were used to measure depression, four different instruments were used to measure anxiety, and three different instruments were used to measure PTSD. The use of different measurement instruments makes direct comparison of the results across studies difficult. Still, all of the studies converge on the same general conclusion and suggest a rather consistent pattern of heightened levels of depression, anxiety, and PTSD among parents after SB.

Additionally, great variability around the mean time since SB was observed in the cross-sectional studies [3, 35, 37]. Thus, the studies preclude inferences regarding the development of mental health over time among parents who experienced SB.

The longitudinal studies found symptoms of depression, anxiety, and PTSD to be at its highest the closer to the stillbirth they were measured, with a declining trajectory with time [2, 5, 36]. These results suggest that for some, SB may perhaps best be characterized as an acute stressor temporarily impacting their mental health. For others, however, such symptoms could be long lasting. It should be noted that although a higher risk of depression and anxiety was also observed in the SB group at later follow up, the estimates from these longitudinal studies had large confidence intervals, suggesting high uncertainty in the point estimates. Based on the reported confidence intervals, the difference between the SB group and controls further appeared to not be significantly different after 8 months and at later follow ups for depression, and at the 30-months follow-up for anxiety. As such, caution should be applied when interpreting these results, and calls for larger scale studies that may more robustly examine long-term symptoms of depression, anxiety, and PTSD after SB. Furthermore, the longest follow-up time in these studies was 30 months. Thus, future studies with longer follow-ups are needed in order to illuminate how symptoms persist and develop after this.

Moreover, to identify for whom and under which circumstances SB may be associated with prolonged symptoms, studies examining moderators are warranted. As identified in the present review, social support, marital status, and cognitive factors could be potential candidates.

### Predictors

#### Social and professional support

Three studies [3, 36, 43] found social and professional support to influence the association between SB and depression, anxiety, and PTSD. None of these studies included men in their sample. Thus, little is known about the association between social support and paternal mental health. For women, both perceived professional help and support from family and friends were of importance for mental health. In the study by Surkan et al. [43], marital status was the strongest predictor of maternal depressive symptoms when analyzing other demographic variables such as education, employment, religion, and age. This indicates being married as a strong protective factor of depressive symptoms after SB. A possible explanation for this is that women with a supportive spouse may perceive more social support to accommodate thoughts and feelings after losing a child [43], thus buffering against the loneliness felt by the potential lack of support and understanding from other family members or friends [13]. Marriage could, therefore, be considered an aspect of social support. Interestingly, the study that found the lowest risk of depression across all studies in the present review investigated only married participants who had experienced SB compared with married women who experienced live birth [39]. Hence, it appears important to consider marital status when assessing and comparing studies investigating SB.

#### Healthcare

Various aspects of bereavement care could affect maternal adjustment after SB. In the present review, two studies found that seeing the stillborn child for as long as wanted could be beneficial for mothers’ mental health [40, 42]. This implies that healthcare workers should assess the mothers’ wishes to see the stillborn, and if deemed appropriate, facilitate and encourage them to follow these wishes. Healthcare workers could also encourage the mother to collect concrete tokens of remembrance, as elevated risks of depression and anxiety were found in women who had not collected such items [40, 42].

Findings were uncertain regarding the association between holding the baby after SB and parental mental health. Chung and Reed [35] did not find a significant association, whereas Råadestad et al. [41] found a potential beneficial effect, but the small sample size in this study yielded high uncertainty in the estimate, precluding clear conclusions to be reached. Inconclusive findings are in accordance with previous systematic reviews on the effect of holding versus not holding the stillborn [28, 49]. This may suggest variability in how parents react to holding the stillborn and that health care providers should respect individual preferences, a point also identified in a qualitative synthesis of qualitative studies on parents’ experience following SB [50]. Further research is needed to detail for whom and under what circumstances holding the stillborn may be beneficial to provide common guidelines based on empirical findings in bereavement care.

In a survey conducted for the Lancet stillbirth series in 2016, it was found that women who experienced SB felt stigmatized, socially isolated, and less valued by society [51]. These findings align with some of the predictors identified in the present review. For instance, maladaptive cognitions about the self or the world were associated with more symptoms of depression [37] and PTSD [36]. Negative self-view was further associated with comorbidity in mental health problems [35]. As such, information about the link between negative cognitions and symptom severity may be useful for health personnel providing services to parents following SB.

#### Limitations of the literature

Several limitations in the literature were identified. First, many studies to date have combined data on different forms of perinatal loss, including miscarriage (early pregnancy loss), SB (late pregnancy loss), and neonatal death (0 to 28 days after birth). Different types of perinatal loss may affect parents in different ways [2, 4, 5, 52]. Combining them limits the possibility of detecting potential unique mechanisms that are linked to parents’ mental health outcomes. This also led to many studies being excluded from the present review. Knowledge of potential differences in mental health after a miscarriage, SB, and neonatal death may aid the development of healthcare guidelines for parents that are tailored for their type of loss.

Second, various measures were used to assess depression, anxiety, and PTSD. This makes aggregation and comparison of individual effect sizes across studies challenging. Third, the studies on anxiety following SB primarily used broad measures that fail to differentiate between anxiety subcategories. To our knowledge, only one study [53] has investigated specific forms of anxiety after perinatal loss and found that bereaved women had more than twice the odds for generalized anxiety disorder and social phobia compared to controls. Hence, identifying subcategories of disorders could be essential to enhance good clinical practice to support bereaved parents [53].

Fourth, although a comprehensive search strategy was employed, only one study on OCD following SB was identified [53]. The study found that SB may also be associated with OCD, but the study did not meet our inclusion criteria, as different types of perinatal loss were combined. As traumatic experiences, life transitions, and pregnancy or birth complications are considered risk factors for developing OCD [54, 55], more research is warranted to clarify the association between SB and OCD.

Furthermore, the majority of studies utilized a cross-sectional design and mean time since SB varied greatly. The longitudinal studies identified had small samples, which induces high uncertainty in the presented estimates. Although we acknowledge the challenge of conducting larger scale longitudinal studies on this topic, such studies are needed in order to more robustly examine long-term outcomes in parents following SB. Thus, absence of longitudinal studies investigating long-term consequences in parents after SB is a general problem within this research field [56].

Additionally, with one notable exception [38], baseline measures of mental health (before the event of SB) were not available in the included studies. A history of mental health problems is considered one of many risk factors of SB [57]. For example, one study [58] found an elevated risk for SB in women with a history of mental health problems compared to controls with no previous mental illness. This suggests a possible bi-directional relationship between SB and parental mental health. As such, elevated levels of mental health problems might already be present among some parents before the SB event. To gain more knowledge of these processes, future studies could track respondents from before to after the event of SB. Still, we acknowledge the methodological challenges of designing and conducting such studies.

Another related weakness is the general lack of information about other adverse life events that could co-occur with SB experiences and symptoms of parental mental health problems. Since several studies measure depression, anxiety, and PTSD years after the SB, it is uncertain whether the symptoms are solely due to the SB or other potential adverse life events.

Although the present review identified 13 studies eligible for inclusion, several of the studies were conducted by the same authors using the same data source. Four of the studies [40–43] had a sample of women who experienced SB in 1991, identified through the medical birth register of Sweden and three of the studies investigated participants who experienced SB, NDD, or SIDS in Queensland between 1985 and 1988 [2, 4, 5]. Therefore, the overall findings in the present review might be prone to common source bias, which also limits the generalizability of the findings. We included studies utilizing the same data source because they investigated different predictors of depression and anxiety, and because they were partly based on different sub-samples. For example, Boyle et al. [2] included only women in the longitudinal study, while Thearle et al. [4] and Vance et al. [5] included both mothers and fathers in their sample. Also, the studies used different designs, providing results of depression and anxiety at various points of follow-up. Furthermore, only Vance et al. [5] and Boyle et al. [2] included men in their samples.

Of the 13 articles in the present review, ten studies focused exclusively on women. This is highlighted as a common trend within this field [14, 25] and prevents the current review from drawing firm conclusions about potential gender differences in mental health outcomes after SB. For example, none of the identified studies on PTSD included men in their samples. The few studies investigating men’s reactions after SB suggest that paternal and maternal reactions might differ [10, 59]. Men could have other coping strategies than women and might benefit from other interventions. For example, men tend to exhibit avoidance behavior to a greater extent than women [25] and can have difficulties in reaching out to receive help from support services [59, 60]. Increased knowledge of men’s reactions to SB could provide valuable information for health personnel, family, and friends on how to support men experiencing SB.

Lastly, the majority of the articles identified in this review (*n* = 12) were conducted in Western, Educated, Industrialized, Rich, and Democratic (WEIRD) societies [61]. This limits the ability to generalize the findings to countries with other cultural, economic, and medical practices. For example, access to health care may be more variable in LMICs. If this is the case, parents who experienced SB in these countries could lack professional support and follow up, which in turn could lead to a greater negative impact on parental mental health than identified in this review [62]. It is also important to note that the quality of postpartum healthcare likely differs between high-income countries as well, and the results of the present review do not necessarily generalize across all these countries.

#### Strengths and limitations of this review

A notable strength of the present review was the implementation of a comprehensive and systematic search strategy and the application of clearly defined inclusion and exclusion criteria. All identified studies were further screened independently by two researchers. Another strength was the inclusion of five studies published between 2015 and 2020 that have not been included in previous reviews [35–39], thus providing updated knowledge of the phenomenon.

Some limitations of the present review should still be noted. Firstly, our search strategy was limited to English-language articles identified in PsychINFO and Web of Science. Thus, potential articles written in other languages or not found in these two databases that otherwise met our inclusion criteria were omitted. It is also possible that our search strategy failed to detect other English-written eligible articles. Given our broad search strategy with many hits, we do, however, not believe that this poses a serious threat to the findings reported in this review. However, due to the few identified articles meeting our inclusion criteria, all were included regardless of potential methodological weaknesses, resulting in studies of varying quality. Some of the included studies also utilized the same data, which might introduce common source bias. Still, as the overall trend of the links between SB and parental mental health was similar across studies using unique data, this does not appear to have impacted the major findings of the present review. Another potential limitation was that articles solely focusing on grief as the primary outcome were excluded, as grief and depression may co-occur [63]. Still, although grief and depression measures often have symptoms that overlap (e.g., sadness), the two phenomena are still considered two distinct psychological constructs [64]. To isolate and provide a detailed overview of depression following SB, we excluded studies on grief.

Lastly, we excluded 22 otherwise eligible studies that included women experiencing SB after multiple births or pregnant women who had previously experienced SB. There may, however, be important differences between various types of SB experiences that may impact the parent’s mental health. As we sought to distill the links between SB and mental health among women with singleton birth, these were therefore excluded.

## Conclusion

To conclude, the present review finds that parents who experience SB display a considerably higher risk of experiencing symptoms of depression, anxiety, and PTSD compared to parents with live births. For some, these symptoms become pathological and long-lasting. Especially unmarried women with low levels of social support appear prone to mental health problems after SB. Healthcare professionals should also be aware of predictors related to care and management after SB. Preventive actions, such as professional and social support, could facilitate a healthy mourning process and reduce the risk of symptoms becoming pathological and long-lasting. Since the studies found prolonged symptoms, this highlights the importance of continued healthcare follow-up for those at risk.

The current research on depression, anxiety, and PTSD after SB has several potential limitations and gaps that may be targeted in future research. These include assessment with different measurement instruments, a paucity of longitudinal studies, lack of research in LMICs, few studies including men, and several studies grouping together different forms of perinatal loss. In addition, more research on the association between SB and OCD is warranted.

## Supplementary Information

**Additional file 1.**

## Data Availability

Data sharing is not applicable to this article as no datasets were generated or analysed during the current study.

## Abbreviations

SB: Stillbirth
GSA: Gestational age
PTSD: Posttraumatic Stress Disorder
OCD: Obsessive-Compulsive Disorder
LMIC: Low−/middle-income country
CI: Confidence Interval
RR: Relative risk
OR: Odds ratio
