# Supplementary material for: Depression, anxiety, PTSD, and OCD after stillbirth: a systematic review
Source: BMC Pregnancy Childbirth. 2021 Nov 18;21:782. doi: 10.1186/s12884-021-04254-x (PMC8600867; doi:10.1186/s12884-021-04254-x)
Supplement: Supplementary file 1 — Additional file 1. [file 12884_2021_4254_MOESM1_ESM.docx]

**Supplementary Table 1.** Quality assessment of the included studies.

|  | **Criteria** | Boyle [2] | Chung [38] | Crawley [3] | Horsch [45] | Kokou [43] | Lewkowitz [37] | Radestad [36] | Radestad [42] | Surkan [40] | Surkan 09 | Surkan, [35] | Thearle [4] | Vance [5] |
| --- | --- | --- | --- | --- | --- | --- | --- | --- | --- | --- | --- | --- | --- | --- |
| 1 | Question/objective sufficiently described? | 2 | 2 | 2 | 1 | 1 | 2 | 1 | 1 | 2 | 2 | 2 | 2 | 1 |
| 2 | Study design evident and appropriate? | 2 | 2 | 2 | 2 | 2 | 2 | 1 | 1 | 1 | 2 | 2 | 2 | 2 |
| 3 | Method of subject/comparison group selection or source of information/input variables described and appropriate? | 2 | 1 | 2 | 1 | 1 | 2 | 2 | 2 | 2 | 2 | 1 | 1 | 1 |
| 4 | Subject (and comparison group, if applicaple) characteristics sufficiently described? | 0 | 2 | 2 | 2 | 2 | 2 | 0 | 2 | 1 | 2 | 1 | 1 | 1 |
| 5 | If interventionl and random allocation was possible, was it described? | N/A | N/A | N/A | N/A | N/A | N/A | N/A | N/A | N/A | N/A | N/A | N/A | N/A |
| 6 | If interventional and blinding of investigators was possible, was it reported? | N/A | N/A | N/A | N/A | N/A | N/A | N/A | N/A | N/A | N/A | N/A | N/A | N/A |
| 7 | If interventional and blinding of subjects was possible, was it reported? | N/A | N/A | N/A | N/A | N/A | N/A | N/A | N/A | N/A | N/A | N/A | N/A | N/A |
| 8 | Outcome and (if applicable) exposure measure(s) well defined and robust to measurement/misclassification of bias? Means of assessment reported? | 2 | 2 | 2 | 2 | 2 | 2 | 2 | 2 | 2 | 1 | 2 | 1 | 2 |
| 9 | Sample size appropriate? | 1 | 1 | 2 | 1 | 1 | 2 | 2 | 2 | 2 | 2 | 2 | 2 | 1 |
| 10 | Analytic methods described/justified and appropriate? | 2 | 2 | 2 | 2 | 2 | 2 | 2 | 2 | 2 | 2 | 2 | 0 | 1 |
| 11 | Some estimate of variance is reported for the main results? | 2 | 2 | 2 | 2 | 2 | 2 | 2 | 2 | 2 | 2 | 2 | 2 | 2 |
| 12 | Controlled for confounding? | 2 | 2 | 0 | 2 | 2 | 2 | 2 | 0 | 2 | 2 | 2 | 1 | 1 |
| 13 | Results reported in sufficient detail? | 2 | 2 | 2 | 2 | 1 | 2 | 1 | 2 | 2 | 2 | 2 | 1 | 2 |
| 14 | Conclusions supported by the results? | 2 | 2 | 2 | 2 | 2 | 2 | 1 | 1 | 2 | 1 | 2 | 0 | 2 |
|  | **Total score/possible maximum score** | 19/22 | 20/22 | 20/22 | 19/22 | 18/22 | 22/22 | 16/22 | 17/22 | 20/22 | 20/22 | 20/22 | 13/22 | 16/22 |
|  | **Quality score** | 0.86 | 0.90 | 0.90 | 0.86 | 0.81 | 1 | 0.72 | 0.77 | 0.90 | 0.90 | 0.90 | 0.59 | 0.72 |
|  | N/A = not applicable, 2 = yes, 1 = partial, 0 = no |  |  |  |  |  |  |  |  |  |  |  |  |  |
